# Process Mining-Supported Emergency Room Process Performance Indicators †

**DOI:** 10.3390/ijerph17176290

**Published:** 2020-08-28

**Authors:** Minsu Cho, Minseok Song, Junhyun Park, Seok-Ran Yeom, Il-Jae Wang, Byung-Kwan Choi

**Affiliations:** 1 Research Institute of Industry & SME Strategy, Korea Institute of Industrial Technology, Seoul 06211, Korea; mcho@kitech.re.kr; 2 Department of Industrial & Management Engineering, Pohang University of Science and Technology, Pohang 37673, Korea; jun0620@postech.ac.kr; 3 Department of Emergency Medicine, Pusan National University Hospital, Busan 49241, Korea; seokrany@pusan.ac.kr (S.-R.Y.); jrmr9933@gmail.com (I.-J.W.); 4 Department of Neurosurgery, Pusan National University Hospital, Busan 49241, Korea; spine@pusan.ac.kr

**Keywords:** process performance indicators, process mining, emergency room, healthcare, performance measurements

## Abstract

Emergency room processes are often exposed to the risk of unexpected factors, and process management based on performance measurements is required due to its connectivity to the quality of care. Regarding this, there have been several attempts to propose a method to analyze the emergency room processes. This paper proposes a framework for process performance indicators utilized in emergency rooms. Based on the devil’s quadrangle, i.e., time, cost, quality, and flexibility, the paper suggests multiple process performance indicators that can be analyzed using clinical event logs and verify them with a thorough discussion with clinical experts in the emergency department. A case study is conducted with the real-life clinical data collected from a tertiary hospital in Korea to validate the proposed method. The case study demonstrated that the proposed indicators are well applied using the clinical data, and the framework is capable of understanding emergency room processes’ performance.

## 1. Introduction

The plethora of technological advancements in healthcare environments has increased enormously in recent years. To this end, existing approaches can be divided into two main research streams, such as biological and engineering development, according to the general goal of improving the quality of care. The biological-oriented research, a direction that is familiar to the public, is the natural science-oriented approach to developing advanced therapeutic methods, including the development of new drugs and medical devices by scientifically understanding human phenomena. The engineering-oriented research tries to analyze and improve the activities of medical stakeholders based on engineering methods such as information technology, optimization, simulation, etc. Such approaches usually emphasize the comprehensive management of clinical processes inevitable for clinical quality control, including process analysis and improvement in the medical environment [1].

Only referring to care provided directly to patients, there are numerous processes in the healthcare context. A typical method is to distinguish clinical processes with the patient type, such as non-elective care (e.g., emergency room patients) and elective care (e.g., outpatients and inpatients) [2]. Here, in general, much effort is attempted for elective processes involving a large number of standard and routine patients. Nevertheless, it is required to manage the emergency room (ER) processes since it is highly complicated in general [3,4]. In addition, it is often overcrowded and out of control since it is exposed to the risk of unexpected factors [5]. For these reasons, comprehensive process management for efficient operation and care quality management is considered to be essential in the ER context.

In systematically managing these processes, referring to the ingredients of business process management, there are several reliable methods such as process modeling, process analysis, and process redesign. Above all, performance measurements are of paramount importance in managing processes [6]. In particular, quantitative performance analysis is getting a keen interest due to the abundance of data and advances of data-driven methods in a healthcare environment [7,8].

Process mining, i.e., a relatively young discipline focused on deriving knowledgeable process-related insights from event logs and known as useful for quantitative performance analysis, has enabled us to perform data-driven process analyses [2]. As such, process mining has yielded many successful applications in the healthcare domain, and there have been previous studies using process mining in the ER environment. For example, [9] proposed the comprehensive question-driven methodology for analyzing the ER processes using process mining with the four schemes, including process discovery, conformance analysis, performance analysis, and organization analysis.

However, there are relatively few works to comprehensively analyze the ER processes by diversifying and specifying the viewpoints of analysis. A couple of researchers have tried to apply concrete perspectives, e.g., time, patients, and resource-utilization [10]. Despite their attempts, it is insufficient to provide details on what aspects of the emergency room will be analyzed for a specific purpose. Furthermore, they do not provide the details about process performance indicators for directly evaluating ER processes, including how to define performance indicators and which data should be collected.

This paper proposes a framework of process performance indicators (PPIs) for ER processes that address the limitations mentioned above. To this end, our framework takes into account the devil’s quadrangle in business process performance analysis, which consists of time, cost, quality, and flexibility [6]. These four perspectives are the primary direction of performance analysis to uncover the insights in ER processes. This study also develops comprehensive performance indicators for evaluating ER process management by extending our previous work suggesting PPIs that can measure the effects of business process redesigns using process mining functionalities [11]. More specifically, we first prepare PPIs that can be analyzed using the clinical event logs and verify them with a thorough discussion with clinical experts in the emergency room. Furthermore, to validate the applicability of our framework, we present a case study result using the real-life clinical data collected from a tertiary hospital in Korea. The case study demonstrated that the proposed indicators are well applied using the clinical data, and the framework is capable of understanding emergency room processes’ performance. In the proposed framework, the devil’s quadrangle, i.e., a qualitative performance measurement tool of process redesign, is quantitatively measured from event logs in the process analysis and monitoring stage. It provides an actionable tool that can lead to process management and decision support and can be used as a cornerstone for the improvement of emergency room processes.

The remainder of this paper is organized as follows. Section 2 summarizes related works on process mining in emergency rooms and process performance measurement. Then, in Section 3, we describe our framework and process performance indicators in detail. Section 4 shows the application of the proposed framework in the case study. Section 5 discusses the result of the case study and the limitations of the proposed method. Finally, Section 6 concludes the paper and describes future directions.

## 2. Related Work

This section provides an overview of existing works relevant to our research. We first describe process mining research applied for exploring emergency room processes in Section 2.1, and then process performance measurement is discussed in Section 2.2, including process performance indicators.

### 2.1. Process Mining in Emergency Rooms

There have been numerous research efforts to apply process mining into a healthcare setting with the different process types, such as non-elective and elective cares [12]. These studies have significantly contributed to improving medical quality by understanding and improving healthcare processes. Among them, a subset of efforts have focused on solving the problems involved in emergency room processes using process mining techniques. In summary, limited to medical treatment processes (i.e., excluding organizational processes), we can classify these works into three directions: proposing a framework for analyzing ER processes, suggesting a couple of metrics for measuring the ER performances, and applications with ad-hoc approaches.

Several articles proposed a series of steps to delve into the ER processes, i.e., a framework or methodology. In [9], the authors proposed a comprehensive question-driven methodology, which provides a data reference model, frequently-posed questions, and the detailed stages to solve the questions using process mining. [13] proposed the six-phase method for performance analysis of emergency room episodes: extraction and transformation, activity aggregation, filtering, discovery, analysis, and results and evaluation. [10] presented a methodology, including six phases: identification of performance indicators, data collection and preparation, process discovery, process monitoring, process analysis, and performance comparison. [14] suggested a methodology to verify compliance with clinical guidelines for actual clinical practice using process mining. As far as the second research direction is concerned, [15] suggested a couple of time-related metrics using process mining tools. In addition, [16] proposed time-related indicators that focus on the elapsed time between two ER activities, such as triage and doctor seen. In addition to presenting indicators, there have been several applications with the ad-hoc approaches to understand ER processes [17,18,19,20,21,22,23,24,25]. For example, [18] introduced an approach to evaluate the capabilities of process mining to the ER process on the stroke case, and [19] explored the effects of interactive process mining using the value-based technologies. In addition, [22] performed a comparative analysis of four different hospitals by focusing on control-flow and time perspectives, and [23] applied the trace clustering technique for deriving more explicit process maps. In addition, [24] proposed an ad-hoc process mining approach to discover the ER patient paths, and [25] suggested how to derive optimal layout of an emergency room by analyzing the care processes.

These existing studies have devoted to enabling process analysis based on process mining for the healthcare environment, specifically emergency rooms. However, they limited to suggest a framework or a methodology with a holistic view and provided little guidance on defining process performance indicators. In addition, although several articles have covered on offering concrete indicators, their achievements were confined to offering just a couple of time-related indicators.

### 2.2. Process Performance Measurement

Process performance measurement has been getting a keen interest and one of the highly active research disciplines in business process fields. It has been conducted in earnest since the development of Balanced Scorecard [26]. They suggested a tool to measure process-related performances in four perspectives, including financial, customer, internal business, and innovation and learning, and this research has been introduced as a typical example that applies the strategic planning and the management system. On the basis of this study, existing process performance measurement research has been tackled in two main streams: qualitative and quantitative measurement. Regarding the qualitative approach, there was an approach to enhance the balanced scorecard, which proposed a strategy map for building a strategic objective of an organization [27]. In addition, regarding the qualitative performance measurement methods, there have been numerous efforts to effectively assess process performances, including Business Excellence Model, Cambridge Model, Integrated Performance Measurement, and Performance Pyramid [28].

Despite these efforts, it is a fact that quantitative measurement methods are mainly studied in academia and applied in practice in recent years. This is caused by multiple factors, including large amounts of available data, development of data-driven techniques, need for the contraction of human involvement, and need for detailed measurements [6]. In this regard, the previous works can be divided into three research themes: (i) providing a structure, i.e., a system or framework, to measure process performance, (ii) suggesting performance measurements for a specific context, and (iii) a generic approach for PPI modeling.

First, [29] implemented the process performance measurement system, which can identify performances of business processes and compare them with target values. The research included financial, employee, customer, societal, and innovation aspects by explaining the need for the multidimensional system. In addition, [30] proposed a fuzzy cognitive strategy map as an alternative scenario-based strategy map, which overcomes the existing limitations of balanced scorecard and strategy map. In addition, there was a framework for developing a process-based approach to performance measurement system design [31]. Regarding the second research stream, there have been multiple attempts to provide how to measure performances for a specific context. For example, [32] proposed performance measures and metrics available in supply chain management and logistics. [33] presented an analytical model to develop process quality index in automotive business processes. In addition, many applications have been performed in numerous environments, including banking [34], service [35], and semiconductor manufacturing [36]. For the third issue, [37] proposed a method to develop process indicators based on a key performance indicator ontology. [38] suggested an organizational performance indicator modeling framework, which includes ten elements: name, definition, type, timeframe, scale, min-max value, source, owner, threshold, and hardness. In addition, [39] proposed PPINOT, i.e., a metamodel to define PPIs comprehensively, which includes how to connect elements in business processes and PPIs and provides an implementation of the metamodel using description logics.

Unlike the studies reviewed above, our approach proposed concrete process performance indicators with time, cost, quality, and flexibility perspectives. To this end, [11] presented a framework of process performance indicators to assess the effects of business process redesigns. However, it has a limitation of not sufficiently suggesting indicators to be utilized immediately to manage the ER processes using process mining techniques.

## 3. Method

In this section, we explain a framework for defining *emergency room process performance indicators* (*ERPPIs*) for managing emergency room processes. In Section 3.1, we describe the preliminaries, including the devil’s quadrangle and ER clinical event logs utilized in this paper. After that, Section 3.2 will discuss the proposed framework, and Section 3.3, Section 3.4, Section 3.5 and Section 3.6 will provide the details on the defined ERPPIs for the time, cost, quality, and flexibility perspective, respectively.

### 3.1. Preliminaries

#### 3.1.1. Devil’s Quadrangle

We employed the devil’s quadrangle to measure the performance of emergency room processes with various viewpoints. The devil’s quadrangle consisting of time, cost, quality, and flexibility angles is a framework for quantitatively evaluating the effect of business process redesigns. Each perspective aims to assess the time required to handle a process instance, the cost of running the process, the quality of service associated with the process, and the ability to handle variations. Therefore, it is not limited to utilizing for determining the effects of process redesigns, and we can apply it to measure the performance of a specific business process. For this reason, we develop a framework based on this framework. Therefore, it is not limited to being utilized to determine the effectiveness of process redesign and can be applied to measure a specific business process’s performance. For this reason, we develop a framework based on the devil’s quadrangle.

#### 3.1.2. ER Clinical Event Logs

Before explaining PPIs, we formally define the emergency room clinical event logs, i.e., the input of process mining for the ER process analysis. Definition 1 shows events, cases, and event logs.

**Definition** **1** (Event, Case, Event Log)**.** *Let A,O,T,ET be a finite set of activities, originators, timestamps, and event types, respectively. E=A×O×T×ET is the set of events, i.e., combinations of an activity, an originator, a timestamp, and an event type (e.g., $e_{i}=\left\{a_{i},o_{i},t_{i},et_{i}\right\})$. Let L be an event log which has a multiset of traces, and $C=\{c_{1},c_{2},c_{3},\ldots ,c_{k}\}$ be the set of cases. A trace $\sigma_{k}=\left\{e_{k,1},e_{k,2},e_{k,3},\ldots ,e_{k,n}\right\}$ is mapped into a case $c_{k}$, where $e_{k,n}$ denotes n-th event of the k-th case.*

Events and cases can have multiple attributes, including activities, originators, variants, and patient identifiers. Here, unlike other clinical event logs, we can identify that emergency values are assigned to cases. In the early stages of the emergency room process, the triage activity measures the patient’s emergency level as an emergency indicator, e.g., Korean Triage and Acuity Scale (KTAS) [40], and the hospital provides clinical services based on this. They are usually recorded in the ER logs. In addition, we define mapping functions for event and case attributes in Definitions 2 and 3 and activity relations where two events have causal relations in Definition 4.

**Definition** **2** (Mapping functions for event attributes)**.** *Let E,A,O,T,ET be the set of events, activities, originators, timestamps, and event types. Functions $M_{A}\in E\to A$, $M_{O}\in E\to O$, $M_{T}\in E\to T$, and $M_{ET}\in E\to ET$ map each event to the relevant activity, originator, timestamp, and event type, respectively. A function $M_{cost}\in E\to R$ assigns cost values to each event (e.g., $M_{cost}\left(e_{i}\right)$ is the cost of i-th event).*

**Definition** **3** (Mapping functions for case attributes)**.** *Let C be the set of cases, and P be a finite set of patient identifiers. A function $M_{pid}\in C\to P$ assigns patient identifiers to each case. Let $V=\{v_{1},v_{2},v_{3},\ldots ,v_{j}\}$ be a finite set of variants where $v_{j}$ is a nonempty subset of all possible combinations of activities. $M_{var}$ is a function mapping each case to a variant (e.g., $M_{var}\left(c_{k}\right)$ is the variant of k-th case). Let EM={1,2,3,4,5} be a finite set of emergency values. $M_{emer}$ is a function mapping each case to a emergency value (e.g., $M_{emer}\left(c_{k}\right)$ is the emergency value of k-th case).*

**Definition** **4** (Activity relation)**.** *Let A be the set of activities and originators. Activity relation (AR)⊆A×A is a set of activity relations where two events have causal relations (e.g., $ar_{k,ij}=\left\{\left(a_{k,i},a_{k,j}\right)|a_{k,i},a_{k,j}\in A\right\}$ where $e_{k,i}$ is the predecessor of $e_{k,j}$(i.e.,$e_{k,i}>e_{k,j}))$.*

### 3.2. A Framework for Emergency Room Process Performance Indicators

**Figure 1:**
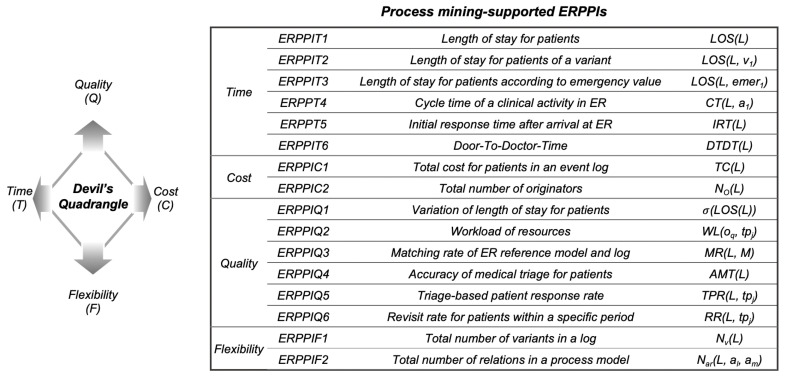
Overview of the proposed framework.

Figure 1 shows an overview of the proposed framework. As described earlier, when developing ERPPI, we use four perspectives that are used mainly for quantitative business process performance analysis. From four perspectives, we proposed 16 performance indicators presented in Figure 1. To define these indicators, we selected candidates process performance indicators that can be derived from clinical event logs using process mining techniques. Clinical experts in the emergency department assessed the usefulness and applicability of the candidates in practice. The remaining section provides a detailed explanation of the ERPPIs proposed in each perspective.

### 3.3. Time-Related ERPPIs

Most organizations aim to manage business processes through improvements in time-related indicators such as processing time and waiting time. In the clinical processes for the emergency room, time-related performance measures are essential since they are highly relevant to the clinical outcomes. This study suggests five indicators in the time perspective: length of stay for patients (*ERPPIT1*), length of stay for patients of a variant (*ERPPIT2*), length of stay for patients according to emergency values (*ERPPIT3*), cycle time of a clinical activity (*ERPPIT4*), initial response time after arrival at ER (*ERPPIT5*), and Door-To-Doctor-Time (*ERPPIT6*). Following are the definitions of time-related process performance indicators (Definitions 5–10).

**Definition** **5** (ERPPIT1: Length of stay for patients)**.** *Let LOS(L) be the length of stay for patients in an event log L.*

*- $LOS\left(L\right)=\left\{t_{k,n}-t_{k,1}|\forall_{0<k\leq |c|}\forall_{0<i\leq n}\;c_{k}\in L\wedge e_{i}\in c_{k}\right\}$*

**Definition** **6** (ERPPIT2: Length of stay for patients of a variant)**.** *Let $LOS(L,v_{1})$ be the length of stay for patients of a variant $v_{1}$ in an event log L.*

*- $LOS\left(L,v_{1}\right)=\left\{t_{k,n}-t_{k,1}|\forall_{0<k\leq |c|}\forall_{0<i\leq n}\;c_{k}\in L\wedge e_{i}\in c_{k}\wedge M_{var}\left(c_{k}\right)=v_{1}\right\}$*

**Definition** **7** (ERPPIT3: Length of stay for patients according to emergency values)**.** *Let $LOS(L,emer_{1})$ be the length of stay for patients according to the emergency values EM={1,2,3,4,5} in an event log L.*

*- $LOS\left(L,emer_{1}\right)=\left\{t_{k,n}-t_{k,1}|\forall_{0<k\leq |c|}\forall_{0<i\leq n}\;c_{k}\in L\wedge e_{i}\in c_{k}\wedge M_{emer}\left(c_{k}\right)=emer_{1}\right\}$*

**Definition** **8** (ERPPIT4: Cycle time of a clinical activity in an event log)**.** *Let $CT(L,a_{1})$ be cycle time of a clinical activity $a_{1}$ in an event log L.*

*- $CT\left(L,a_{1}\right)=\left\{t_{k,i+1}-t_{k,i}|\forall_{0<k\leq |c|}\forall_{0<i\leq n}\;c_{k}\in L\wedge e_{i}\in c_{k}\wedge M_{a}\left(e_{k,i}\right)=a_{1}\right\}$*

**Definition** **9** (ERPPIT5: Initial response time after arrival at ER)**.** *Let IRT(L) be response time for patients in an event log L, and it is defined as the difference between the timestamp of the activity relevant to the entry at ER and its successor. Here, let assume that the entry-related activity is denominated as ‘entry’.*

*- $IRT\left(L\right)=\{t_{k,i+1}-t_{k,i}|\forall_{0<k\leq |c|}\forall_{0<i\leq n}\;c_{k}\in L\wedge e_{i}\in c_{k}\wedge M_{a}\left(e_{k,i}\right)=‘entry’\}$*

**Definition** **10** (ERPPIT6: Door-To-Doctor-Time)**.** *Let DTDT(L) be door to doctor time in an event log L, and it is defined as time difference from the hospital arrival (i.e., entry) to seeing a doctor (i.e., consultation). Here, let us assume that the entry-related and consultation-related activities are denominated as ‘entry’ and ‘consultation’, respectively.*

*- $DTDT\left(L\right)=\{t_{k,j}-t_{k,i}|\forall_{0<k\leq |c|}\forall_{0<i,j\leq n}\;c_{k}\in L\wedge e_{i},e_{j}\in c_{k}\wedge M_{a}\left(e_{k,i}\right)=‘entry’\wedge M_{a}\left(e_{k,j}\right)=‘consultation’\}$*

The first two time-related indicators, i.e., *ERPPIT1* and *ERPPIT2*, are performance indicators for the entire clinical process. In particular, regarding the ERPPIT2, if a hospital is having difficulty managing all kinds of ER processes, it is necessary to manage the critical processes among them. *ERPPIT3* calculates the length of stay according to the patient’s emergency. In general, hospitals spend more time on urgent patients, and the lower the emergency, the sooner the patient leaves the emergency room. Therefore, it can be a crucial indicator for evaluating the performance of the emergency room. *ERPPIT4* measures the performance of crucial clinical activities, including treatment, medical testing, and counseling, where it is essential to avoid bottlenecks in the process. Finally, most hospitals attempt to manage the *golden hour*, which is recommended for caring for urgent patients. Regarding this regulation, we propose two measures that must be managed in the ER: (i) the rapid initiation of clinical services for patients, i.e., *ERPPIT5* and (ii) Door-To-Doctor-Time, i.e., *ERPPIT6*. These two signify the times they take to get the first clinical care and see a physician after the hospital arrival, and they are highly dependent on improving the quality of care. Note that all time-related indicators are combined with aggregation functions, e.g., average($f^{AVG}$), median($f^{MED}$), minimum($f^{MIN}$), and maximum($f^{MAX}$).

### 3.4. Cost-Related ERPPIs

As far as the cost-related PPIs are concerned, we need to consider two kinds of cases in event logs: cost-attached and cost-detached logs. As described in the Definition 2, in the cost-attached logs, cost-related information can be recorded as an event attribute. However, on systems with limited data accumulation, there is a limit to including cost-related records. Therefore, this study suggests two cost-related PPIs for these two kinds of cases.

First, *ERPPIC1* represents the total cost to the ER patient. Similar to the time-related indicators, this metric should be utilized with the aggregation functions to produce more meaningful insights. On the other hand, based on the assumption that all resources are full-time workers, we define an alternative indirect cost-related PPI, i.e., the total number of originators in the log (*ERPPIC2*); therefore, it can be calculated from the generally available clinical event logs. Definitions 11 and 12 show the formal definitions of the cost-related ERPPIs.

**Definition** **11** (ERPPIC1: Total cost for patients in an event log)**.** *Let TC(L) be the total cost for patients in an event log L. Here, $M_{cost}$ is the function mapping each event to the relevant cost.*

*- $TC\left(L\right)=\{\sum_{0<i\leq n}M_{cost}\left(e_{k,i}\right)|\forall_{0<k<|c|}\;c_{k}\in L\}$*

**Definition** **12** (ERPPIC2: Total number of originators)**.** *Let $N_{o}\left(L\right)$ be the total number of originators in an event log L.*

*- $N_{o}\left(L\right)=\sum_{q=1}^{m}\left\{\begin{array}{cc} 1 & \;if\;O_{q}\in \left\{\sum_{0<k\leq |c|}\sum_{0<i\leq n}M_{o}\left(e_{k,i}\right)\right\}\\ 0 & \;otherwise \end{array}\right.$*

### 3.5. Quality-Related ERPPIs

In business process management, quality-related performance analysis can be differentiated as external and internal aspects. More in detail, external quality focuses on customers (e.g., patients), while internal quality is related to the view of process performers. In an emergency room environment, external quality may include patient satisfaction and clinical outcomes (e.g., mortality rate or re-visit rate). Here we propose six indicators of quality perspective, especially five internal indicators and an external one.

The first quality-related indicator, i.e., *ERPPIQ1*, shows how varied the cycle time changes in the emergency clinical process. It identifies whether the process is stable and standardized. Definition 13 presents the formal definition of *ERPPIQ1*.

**Definition** **13** (ERPPIQ1: Variation of length of stay for patients)**.** *Let σ(LOS(L)) be the standard deviation of the length of stay for patients in an event log L. Here, the function $f^{STD}$ returns the standard deviation of the values.*

*- $\sigma \left(LOS\left(L\right)\right)=f^{STD}\left(\left\{t_{k,n}-t_{k,1}|\forall_{0<k\leq |c|}\forall_{0<i\leq n}\;c_{k}\in L\wedge e_{i}\in c_{k}\right\}\right)$*

Secondly, the workload of resource (*ERPPIQ2*) indicates the amount of work done by an originator. Since the emergency room is highly crowded in general, this indicator is essential. Definition 14 shows how to measure the workload of resources. As described in the definition, it requires two kinds of values within a specific time period ($tp_{j}$): number of events started ($o_{q},start,tp_{j}$) and number of events terminated ($o_{q},complete,tp_{j}$) by a specific originator ($o_{q}$). In the initial stage (j=1), the workload simply becomes the difference between the number of started and completed events. From the second period (j>1), it is required to consider the workload of the previous stage ($Workload_{o_{q},tp_{j-1}}$).

**Definition** **14** (ERPPIQ2: Workload of resources)**.** *Let NE∈O×ET×TP→R be a function that computes the number of events from a log L for a given resource $\left(o_{q}\in O\right)$, a type ({start,complete∈ET}), and a time period $\left(tp_{j}\in TP\right)$. $NE(o_{q},start,tp_{j})$ denotes the number of events started by the resource $o_{q}$ within the time period $tp_{j}$. Here, if the event type only holds complete, the complete time of the immediately preceding event in the same case becomes the start time. $NE(o_{q},complete,tp_{j})$ denotes the number of events completed by the resource $o_{q}$ within the time period $tp_{j}$. The workload $WL(o_{q},tp_{j})$ for the resource $o_{q}$ within the time period $tp_{j}$ is defined as follows.*

*- $WL\left(o_{q},tp_{j}\right)=\sum_{0<q\leq |m|}\sum_{0<j\leq |p|}\left\{\begin{array}{cc} NE\left(o_{q},start,tp_{j}\right)-NE\left(o_{q},complete,tp_{j}\right) & \;if\;j=1\\ Workload_{o_{q},tp_{j-1}} & \\ \;\;+NE\left(o_{q},start,tp_{j}\right)-NE\left(o_{q},complete,tp_{j}\right) & \;otherwise \end{array}\right.$*

*ERPPIQ3* aims to compare the reference model for the emergency room process and its log. The reference model is a standard process model to be followed in the emergency room. The concept of *conformance checking* is used in comparison. Here, we utilize the *earth mover’s distance and stochastics* [41], and Definition 15 indicates how to measure the matching rate.

**Definition** **15** (ERPPIQ3: The matching rate between ER reference model and log)**.** *Let MR(L,M) be the matching rate that compares between the ER reference model M and the log L.*

*- $MR\left(L,M\right)=1-\sum_{\sigma \in L}\sum_{\sigma^{\prime }\in M}\;$ reallocated$\left(\sigma ,\sigma^{\prime }\right)$ distance$\left(\sigma ,\sigma^{\prime }\right)$*

In operating emergency rooms, it is required to determine the urgency of patients accurately. The triage activity, i.e., determining emergency values according to the patient’s conditions, is performed by care providers or first-aiders. To this end, the relevant health organizations provide a series of rules to determine the emergency values and recommend to follow this. Therefore, it is essential to identify whether the patient’s urgency values follow the pre-defined rules or not, and *ERPPIQ4* indicates this measurement.

In operating emergency rooms, it is required to determine the urgency of patients accurately. Screening activities, that is, determining the level of emergency based on the patient’s condition, are performed by the care provider or first aid. To this end, the relevant health institution recommends providing a set of rules for determining the emergency level and following it. The triage activity, i.e., determining the level of emergency based on the patient’s condition, is performed by care providers or first-aiders. For this purpose, a set of rules for determining the emergency level are provided and recommended to be followed. Therefore, it is essential to identify whether the patient’s urgency values follow the pre-defined rules or not, and *ERPPIQ4* indicates this measurement.

**Definition** **16** (ERPPIQ4: Accuracy of medical triage for patients)**.** *Let $C_{X\Rightarrow \{1,2,3,4,5\}}\left(c_{k}\right)$ denote the classifier to produce the emergency value of a case $c_{k}\in C$, i.e., 1,2,3,4,5, from X variables. $M_{emer}\left(c_{k}\right)$ signifies the function mapping a case $c_{k}\in C$ to the emergency value. Then, accuracy of medical triage for patients AMT(L) is formally defined as follows.*

*- $AMT\left(L\right)=\frac{\sum_{0<k<|c|}\left\{\begin{array}{cc} 1 & \;if\;c_{k}\in L\wedge C_{X\Rightarrow \{1,2,3,4,5\}}\left(c_{k}\right)=M_{emer}\left(c_{k}\right)\\ 0 & \;otherwise \end{array}\right.}{\left|c\right|}$*

The fifth indicator of the quality perspective, i.e., *ERPPIQ5* depends on the fundamental policy that a higher emergency of the patient acquires a more top priority. In this paper, we suggest the triage-based patient response rate by considering two strategies: (1) first come, first served (FCFS), and (2) more urgent people are allowed to be treated early even if they are late. Definition 17 presents the formal explanation of this indicator.

**Definition** **17** (ERPPIQ5: Triage-based patient response rate)**.** *Let $M_{emer}\left(c_{k}\right)$ denote the emergency degree of a case $c_{k}\in C$ in an event log L. Assume that all cases $c_{k}\in C$ are sorted by the completed time of their first event $t_{k,1}$. Then, triage-based patient response rate $TPR(L,tp_{j})$ for a specific time period $tp_{j}\in TP$ is formally defined as follows.*

*- $TPR\left(L,tp_{j}\right)=1-\frac{\sum_{0<k\leq |c|}\left\{\begin{array}{cc} 1 & \;if\;t_{k,2}>t_{k+1,2}\wedge M_{emer}\left(c_{k}\right)>M_{emer}\left(c_{k+1}\right)\\ & \;\wedge f^{MIN}\left(tp_{j}\right)\leq t_{k,2},t_{k+1,2}\leq f^{MAX}\left(tp_{j}\right)\\ 0 & \;otherwise \end{array}\right.}{|c|-1}$*

The last indicator to measure the quality perspective, i.e., *ERPPIQ6* is the revisit rate for patients, which checks the external quality of ER processes. In general, when the ER process is terminated, it transfers to discharge or hospitalization; thus, staying in hospitals is recommended if the patient’s condition is not improved. Thus returning to the emergency room within a specific period signifies that the relevant process has a problematic issue. To this end, this research proposes the appropriate indicator as formally defined in Definition 18. It is necessary to determine the parameter that designates the revisit at emergency rooms, i.e., a specific period $tp_{j}\in TP$.

**Definition** **18** (ERPPIQ6: Revisit rate for patients within a specific period)**.** *Let $RR(L,p_{1})$ denote the revisit rate for patients within a specific time period $tp_{j}\in TP$ in an event log L. Here, $M_{pid}\left(c_{k}\right)$ is the function mapping a case $c_{k}\in C$ to the corresponding patient ID.*

*- $RR\left(L,tp_{j}\right)=\frac{\sum_{0<k<l\leq |c|}\left\{\begin{array}{cc} 1 & \;if\;c_{k},c_{l}\in L\wedge t_{l}-t_{k}\leq tp_{j}\wedge M_{pid}\left(c_{k}\right)=M_{pid}\left(c_{l}\right)\\ 0 & \;otherwise \end{array}\right.}{\sum_{r=1}^{o}\left\{\begin{array}{cc} 1 & \;if\;p_{r}\in \left\{\sum_{0<k<|c|}M_{pid}\left(c_{k}\right)\right\}\\ 0 & \;otherwise \end{array}\right.}$*

### 3.6. Flexibility-Related ERPPIs

To assess the flexibility of emergency room clinical processes, we introduce two indicators, i.e., *ERPPIF1* and *ERPPIF2*, which evaluate whether a process can react to changes. Definition 19–20 provide the formal explanation of each flexibility-related indicator.

**Definition** **19** (ERPPIF1: Total number of variants in a log)**.** *Let $N_{v}\left(L\right)$ be the total number of variants in a log L.*

*- $N_{v}\left(L\right)=\sum_{r=1}^{o}\left\{\begin{array}{cc} 1 & \;if\;v_{r}\in \left\{\sum_{0<k<|c|}M_{var}\left(c_{k}\right)\right\}\\ 0 & \;otherwise \end{array}\right.$*

**Definition** **20** (ERPPIF2: Total number of relations in a process model)**.** *Let $N_{ar}\left(L,a_{l},a_{m}\right)$ be the total number of relations between two activities $a_{l},a_{m}\in A$ in a process model produced by an event log L.*

*- $N_{ar}\left(L,a_{l},a_{m}\right)=\sum_{0<k\leq |c|}\sum_{0<i<j\leq n}\left\{\begin{array}{cc} 1 & \;if\;c_{k}\in L\wedge e_{k,i},e_{k,j}\in c_{k}\wedge a_{l},a_{m}\in A\wedge e_{k,i}>e_{k,j}\\ & \;\;\wedge a_{k,i}=a_{l}\wedge a_{k,j}=a_{m}\\ 0 & \;otherwise \end{array}\right.$*

These two indicators signify to identify whether the process model has an ability to handle a higher variety of cases with different control-flows. More in detail, ERPPIF2 identifies the direct-follows relations between two events, i.e., $e_{k,i}>e_{k,j}$. All these indicators can be described that the ER process can handle patients more flexibly as these values higher.

## 4. Case Study

To demonstrate the applicability of the proposed process performance indicators, we performed a case study with the real-life emergency rooms clinical data collected from the electronic health records (EHR) system in a tertiary hospital in Korea.

### 4.1. Context

In the case study, we collected the clinical event log of the ER patients during 2018. The event log was established in a text (comma-separated values) format, which includes the case identifier, activity, timestamp, and other information (e.g., type and resource), as depicted in Table 1. In addition, it contained 15 medical tasks: entry, basic treatment, first aid treatment, other treatment, diagnostic test, visual test, consultation, cooperation request, cooperation arrival, the decision on hospitalization or discharge, prescription request, prescription receiving, certificate issuing, discharge, and hospitalization.

Regarding the collected event log, we exploited multiple data preprocessing methods for better application. To this end, we investigated the event log according to the data imperfection patterns suggested by [42]. First, in the healthcare information system, events have been collected based on patient identifiers. This case study attempted to analyze the ER process by defining process instances as a series of events from arriving at emergency rooms to going to the home of patients. Nevertheless, a couple of patients had the records that visit multiple times at the ER in the same day. As such, we were not able to use patient identifiers as cases, i.e., *exclusive case*; thus, the combination of patient identifiers and timestamps of their visits were utilized instead of it. In addition, there were records of clinical activities before arriving at the ER, i.e., *inadvertent time travel*. For example, the timestamp of diagnostic tests was ahead of the arrival at ER. In addition, in the event log, multiple events for a specific activity were recorded at the same time, i.e., *form-based event capture*. As a solution to these two types of imperfections, we selected the deletion as a remedy since those appeared only in a few patients. Lastly, there were duplications of the same event for a specific patient, i.e., *collateral events*. In this regard, this was preprocessed by retaining only one of the duplicate events and deleting the rest.

By doing so, we identified that around 460,000 events were included for about 30,000 patients who visited the emergency room in the log. Table 1 provides the snippet of the event log utilized in the case study. In the table, we need to identify the existence of an emergency level. As introduced in Section 3.5, each patient has determined the level of urgency from the medical triage activity. It employs the KTAS that consists of 1 to 5; specifically, the lower the figure, the more urgent. The log also included other attributes of patients, such as age, test results, diagnosis results, etc. As tools, to measure the PPIs, we used the Fluxicon Disco [43], ProDiscovery [44], and ProM tool [45].

### 4.2. Results

#### 4.2.1. Time Perspective

For the time perspective, we applied all proposed indicators to the collected event log. Table 2 presents the results for the performance analysis on ERPPIs.

**Figure 2:**
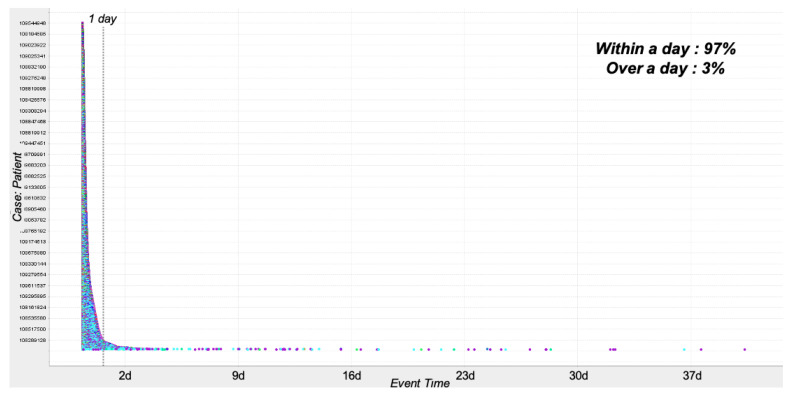
The length of stay analysis result using the dotted chart.

Regarding the length of stays for ER patients, i.e., *ERPPIT1*, we identified that patients stayed 8.4 h in the ER on average, while the median is 4.8 h. In more detail, we attempted to determine the distribution of the length of stays of patients, and Figure 2 presents the result of this. In the figure, we can identify that most of the patients, i.e., 97%, stayed just within a single day, while only 3% of patients stayed over a day. Interestingly, we were able to find a couple of patients who remain for around 40 days.

Concerning the second time-related indicator, i.e., *ERPPIT2*, there was a significant difference of variants according to its characteristic on whether it is connected to hospitalization or discharge. More in detail, inpatients tended to stay longer than discharged patients (hospitalized: 11.5 h and discharged: 5.4 h on average).

After that, regarding the third indicator in the time perspective, i.e., *ERPPIT3*, we calculated the length of stay for patients according to the emergency values. As a result, we identified that care-providers spent more time to severe urgent patients than others; specifically, 11.8 h for the critical patients (i.e., 1 or 2 of the emergency value), 8.9 h for the moderate emergency (i.e., 3), and 6.0 h for the mild emergency (i.e., 4 or 5 of the value), on average.

**Figure 3:**
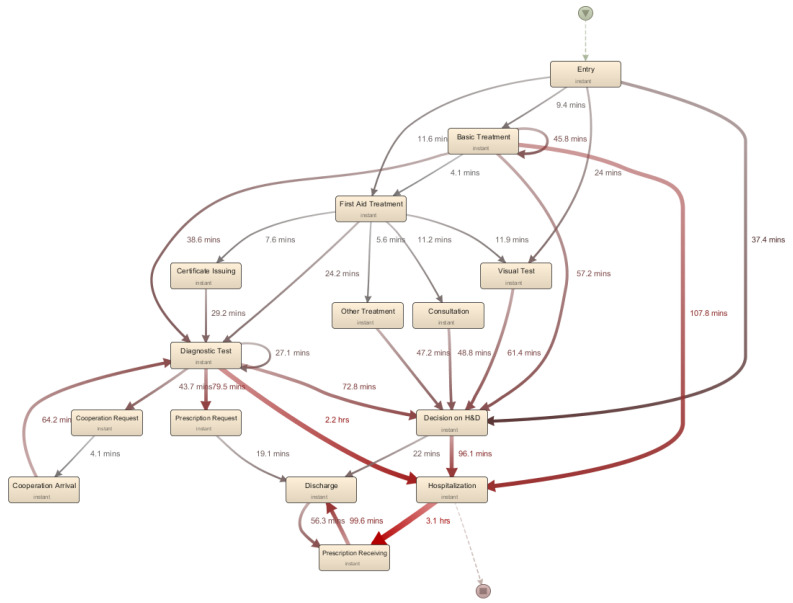
The time-related analysis result with the process model.

We also measured the cycle time for clinical activities in the ER process, i.e., *ERPPIT4*, and Table 2 includes the principal activities in the ER process. Based on the analysis, we identified that several tasks required a relatively long cycle time, including hospitalization and decision on hospitalization or discharge. To identify the preceding activities that cause problematic points, we performed the model-based performance analysis, as depicted in Figure 3. As a result, we identified that it takes a long time from the diagnostic test to hospitalization; besides, it was followed by the decision on hospitalization and discharge to discharge, visual test to prescription request, and diagnostic test to the decision on hospitalization and discharge.

The last performance analysis for the time perspective, i.e., *ERPPIT5* was the initial response time after arrival at the ER of patients. In this regard, it was identified that most patients received the first care services 8.0 min after arrival at the ER; specifically, the measured median and average values were 8.0 and 19.6 min, respectively.

#### 4.2.2. Cost Perspective

**Figure 4:**
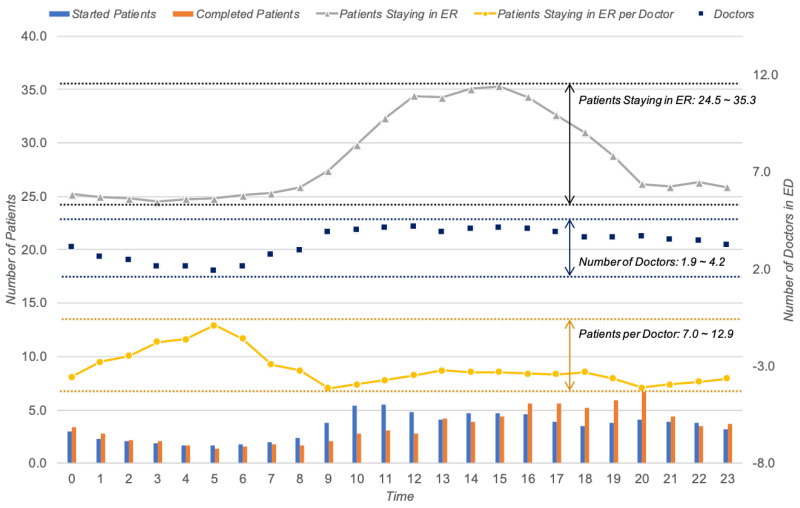
The resource-related analysis result for the cost and quality perspectives.

Regarding the cost perspective, we were not able to apply the first indicator, i.e., *ERPPIC1*, due to the limited records of the event log. In other words, the log utilized in this study did not hold the cost information for each event. As such, instead of it, we measured the number of doctors, i.e., *ERPPIC2*. From the log, we identified that there were 6.8 doctors on average in a single day. More in detail, we identified that it has a time pattern of the value; thus, there was a variation according to time. Figure 4 depicts the resource-related analysis result. In the figure, blue dots and grey lines signify the number of doctors working in the emergency room by each hour and patients staying in the ER, respectively. More in detail, there were 1.9 to 4.2 doctors for every single hour, and they provided the services to 24.5 to 35.3 patients. Here, it was confirmed that there were many medical personnel as the number of patients increased during the daytime.

#### 4.2.3. Quality Perspective

Concerning the quality perspective, we calculated the workloads for doctors in the ER, i.e., *ERPPIQ1*. Due to the limitation of the collected data, the number of patients in the emergency room, rather than the number of medical activities performed by the physician, was assumed as a workload. As a result, we identified that each person averagely managed 11.9 patients in a single day. In addition, there was a variation of workload by each hour, as depicted in Figure 4. More in detail, there was a trend that clinicians were busier at dawn despite the small number of patients, as shown in the yellow line.

Then, the variation of the length of stay for ER patients was computed, i.e., *ERPPIQ2*, and the standard deviation was 8.6 h. More in detail, it had the difference between the variants of patients; specifically, the value of hospitalized patients (10.0 h) was more diverse than discharged patients (5.4 h).

Regarding the *ERPPIQ3*, it was hard to obtain the reference model in this case study; thus, instead of this, we decided to produce the model using the discovery techniques. Although we attempted to measure the matching rate using the stochastic-aware conformance checking [41], we were not able to apply for the ER event log, since the algorithm currently requires an enormous amount of computation. As an alternative, we applied the standard conformance checking algorithm of alignments and the discovered Petri-net using inductive mining [46]. That is, the fitness value was calculated. As a result, we identified that fitness is 0.90; thus, the model well reflected the behaviors in the log.

The fourth quality-related performance indicator, i.e., *ERPPIQ4*, was measured by comparing the records of emergency values in the log and the classified values based on the decision rule proposed by the Ministry of Health and Welfare in Korea. In short, patients get higher emergency value as the lower Glasgow Coma Scale and pulse oxygen saturation, the higher pain score, and blood pressure, pulse rate, respiration rate, and body temperature are away from a specific bound. On the basis of this rule, we identified that the accuracy value is 0.4442 by comparing two values.

**Figure 5:**
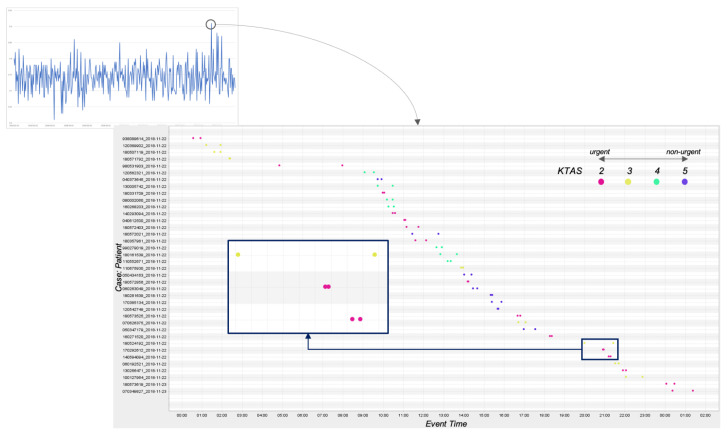
The triage-based patient response analysis result.

For *ERPPIQ5*, the triage-based patient response rate was measured, and the average value was 0.74. To this end, we performed a further analysis with the dotted chart, as depicted in Figure 5. From the chart, we investigated the response-related policies suggested in Section 3.5: (1) first come, first served (FCFS), and (2) more urgent people are allowed to be treated early even if they are late. The figure shows the response pattern of the well-managed day with the high value of 0.91, and the red, yellow, and green and purple dots signify the urgent, moderate, and non-urgent patients, respectively. As presented in the blue box (enlarged) in the figure, we observed that a more urgent person, i.e., the red dots, was treated faster than a less urgent person, i.e., the yellow dots (i.e., the second policy). In addition, it was identified that the hospital follows well the FCFS policy from the second and third cases in the box.

Regarding the last indicator in the quality perspective, i.e., *ERPPIQ6*, we analyzed the revisit rate on the same day (i.e., $tp_{j}=1d$). As a result, it was identified that only 0.38% of patients revisit the hospital.

#### 4.2.4. Flexibility Perspective

Lastly, in the flexibility perspective, we identified 25,004 variants in the log (i.e., *ERPPIF1*). Thus, considering the total number of patients, i.e., 30,000 patients, it was confirmed that most people have different variants from each other. Then, we produced the ER process model with a discovery technique, i.e., frequency mining [47], and measured the number of relations in the model, i.e., *ERPPIF2*. As a result, there were 188 activity relations in the discovered model.

## 5. Discussion

### 5.1. Discussion with ER Experts

To validate the case study result presented in Section 4, we discussed the findings with ER experts. We explained the framework, proposed ERPPIs, and the results to the group of ER medical staff. By doing so, we assessed the overall state of the ER and identified the rationales of them.

First, the hospital has well managed the time-related indicators. Notably, it was confirmed that a couple of points that take a long time. However, taking a long time is inevitable since night admission is prohibited for the convenience of other inpatients. In addition, the rest of the other indicators were within the acceptable range. Regarding the cost perspective, we were not able to measure the direct cost-related indicators due to the issue of limited data access. However, the indirect cost indicator, i.e., the number of clinicians, was maintained at the appropriate level. In terms of the quality, variation of the length of stay and the response to emergency patients were satisfactory. Still, the proper allocation of the work, considering the number of patients was necessary. Finally, we confirmed that the emergency room was operated with sufficient flexibility to care for various patients.

### 5.2. Further Applications of ERPPIs

In the case study, we provided the applications of measuring ERPPIs based on the clinical event log for emergency room processes. This section discusses the further applications of ERPPIs, not just measuring them.

In [46], the author proposed the refined process mining framework, which includes 10 process mining-related activities for three categories (i.e., *cartography*, *auditing*, and *navigation*) that can be applied using historical and current data. Among them, most of the ERPPIs presented in this study is involved in *cartography* and *auditing*, and they use *discover*, *enhance*, *diagnose*, and *compare* activities. However, these indicators are also available for *explore*, *predict*, and *recommend* in navigation, which many researchers are interested in recently. Here, as an example, we introduce the application of navigation for the accuracy of triage, i.e., ERPPIQ4. As described before, classifying the emergency degree of patients with reasonable accuracy is essential to determine the response order for ER patients and their treatment procedures. In most hospitals, triage is performed by the opinions of doctors or first-aiders based on the rules defined by the relevant health organizations. In this regard, it enables more accurate triage as employing data-driven approaches, including machine learning and deep learning techniques. As such, we present the results of its applications.

Summarizing how to build classifiers for emergence degrees, we employed multiple independent variables, such as Glasgow Coma Scale, pulse oxygen saturation, blood pressure, pulse rate, respiration rate, body temperature, pain score, and entry type, and the five-scale emergency score of KTAS was utilized as the dependent variable. In addition, we applied five different classification methods, including a regression-based classifier, decision tree, random forest, support vector machine-based classifier, and multi-perceptron neural net. For the evaluation, we partitioned the data to 70% for the training set and 30% for the test set. Table 3 describes the classification results.

As a result of building classifiers with different methods, we identified that the accuracy of 0.4442 could be improved to 0.7607 by applying the multi-perceptron neural net. Here, this classifier can be utilized not only to enhance the quality of care based on contributing to the accurate determination of the patient’s emergency but also to enable task automation of triage and create a plan for the efficient operation of emergency rooms. Based on this, we argue that ERPPIs can be used for further analysis, such as prediction and real-time monitoring, beyond simple measurement.

### 5.3. Contributions and Limitations

One of the main contributions of this study is to propose process performance indicators that can be used for directly evaluating the ER processes. Unlike existing studies that suggest measurements at a higher level, such as time, cost, and financial perspectives or propose general frameworks, which consist of data preparation, preprocessing, analysis, and evaluation, this study is capable of the application in practice based on the process mining functionality. As such, we believe that the results can become a solid basis for decision making in the ER. In addition, as described in Section 5, our work has an ample possibility of extending to *navigation*, including *prediction* and *recommendation*, beyond merely measuring performance. In other words, it will cover not only analysis but also redesign and monitoring in the business process management lifecycle.

Our work also has several limitations. As far as the case study is concerned, this paper did not cover the whole ERPPIs suggested in Section 3. As described in Section 4.1, the event log utilized in the case study only included the limited attributes of ER patient-related attributes; thus, only a part of them was measured. In addition, this paper provided a single case study. As such, it is necessary to apply our framework to multiple ER event logs to validate it. Regarding the suggested method, it could be extended considering various aspects of the context in the emergency room, as well as aspects that can take advantage of the advanced techniques of process mining. In this study, the opinions of medical experts were collected to evaluate the proposed analysis method. However, the criteria for objectively assessing results are needed. Furthermore, how to improve the ER process based on the ERPPIs measurement is missing. To this end, it can be required for connecting ERPPIs measurements and techniques for business process improvements, such as optimization or simulation. Lastly, we need to provide a tool for the efficient application of the proposed framework and ERPPIs measurement.

## 6. Conclusions

This paper aimed to provide a guideline for measuring performances of emergency room processes in detail. As such, we proposed a framework of Emergency Room Process Performance Indicators (ERPPIs) to assess emergency room processes based on four perspectives: time, cost, quality, and flexibility. More specifically, the framework included 16 indicators that can measure the emergency room performances using process mining functionalities. To validate our approach, we introduced a case study that applies the real-life clinical event log collected from a tertiary hospital in Korea. By doing so, we demonstrated that the indicators presented in this study are well applied using the clinical data, and the framework is capable of understanding emergency room processes’ performance. We also proved that our result could be utilized for further analysis, such as prediction and real-time monitoring, beyond simple measurement, by providing a classifier for emergency degrees.

As future works, we will implement a tool to support the application of the proposed framework, and it will play a key role in applying it in practice. The presented method will also be extended to offering more indicators based on the context or advanced techniques and making a connection of this research to the process redesign that shows improvement plans according to the performance analysis results. Lastly, we only covered a single case study with the application of a subset of indicators; thus, more case studies in different hospitals will be conducted to validate the applicability of our work.

## Figures and Tables

**Table 1:** A snippet of the emergency room (ER) event log.

CaseID	Activity	Timestamp	Type	Resource	Emergency	PatientID
1	Entry	01/02/2018 19:05	Complete	John	3	P1
1	Treatment	01/02/2018 19:17	Complete	Jane	3	P1
1	Consultation	01/02/2018 19:20	Start	Tom	3	P1
2	Entry	01/02/2018 19:21	Complete	John	1	P2
2	Treatment	01/02/2018 19:36	Complete	Jane	1	P2

**Table 2:** Results for performance analysis applying ERPPITs.

ERPPI	Type	Average	Median
ERPPIT1 (LOS(L))	–	8.4 h	4.8 h
ERPPIT2 ($LOS(L,v_{1})$)	*length of stay with hospitalization*	11.5 h	7.5 h
	*length of stay with discharge*	5.4 h	3.4 h
ERPPIT3 ($LOS(L,emer_{1})$)	*Severe* (i.e., 1 and 2 of KTAS)	11.8 h	6.6 h
	*Moderate* (i.e., 3 of KTAS)	8.9 h	5.3 h
	*Mild* (i.e., 4 and 5 of KTAS)	6.0 h	3.5 h
ERPPIT4 ($CT(L,a_{1})$)	*Consultation*	17.2 m	8.0 m
	*Basic Treatment*	27.2 m	6.0 m
	*First-aid Treatment*	9.4 m	3.0 m
	*Diagnostic Test*	10.5 m	2.0 m
	*Decision on Hospitalization or Discharge*	50.7 m	24.0 m
	*Discharge*	27.1 m	5.0 m
	*Hostpitalization*	114.5 m	68.0 m
ERPPIT5 (IRT(L))	–	8.0 m	19.6 m

**Table 3:** Classification results for emergency degrees of ER patients.

Classification Method	Accuracy	Precision	Recall	F1 Score
Baseline (Rule-based)	0.4442	0.4400	0.3741	0.3329
Regression-based Classifier	0.7056	0.6985	0.5997	0.6370
Decision Tree	0.7578	0.7236	0.6158	0.6509
Random Forest	0.7509	0.7749	0.5580	0.6122
SVM-based Classifier	0.6654	0.5685	0.5905	0.5761
Multi-Perceptron Neural Net	0.7607	0.7548	0.6430	0.6812
